# A Crack Size Quantification Method Using High-Resolution Lamb Waves

**DOI:** 10.3390/s21206941

**Published:** 2021-10-19

**Authors:** Xianjun Li, Jinsong Yang, Guangdong Zhang

**Affiliations:** School of Traffic and Transportation Engineering, Central South University, Changsha 410075, China; xianjunli@csu.edu.cn (X.L.); guangdongzhang1995@163.com (G.Z.)

**Keywords:** Lamb waves, crack size quantification, pulse compression, excitation waveform design

## Abstract

Traditional tone burst excitation cannot attain a high output resolution, due to the time duration. The received signal is much longer than that of excitation during the propagation, which can increase the difficulty of signal processing, and reduce the resolution. Therefore, it is of significant interest to develop a general methodology for crack quantification through the optimal design of the excitation waveform and signal-processing methods. This paper presents a new crack size quantification method based on high-resolution Lamb waves. The linear chirp (L-Chirp) signal and Golay complementary code (GCC) signal are used as Lamb wave excitation signals. After dispersion removal, these excitation waveforms, based on pulse compression, can effectively improve the inspection resolution in plate-like structures. A series of simulations of both healthy plates and plates with different crack sizes are performed by Abaqus CAE, using different excitation waveforms. The first wave package of the S_0_ mode after pulse compression is chosen to extract the damage features. A multivariate regression model is proposed to correlate the damage features to the crack size. The effectiveness of the proposed crack size quantification method is verified by a comparison with tone burst excitation, and the accuracy of the crack size quantification method is verified by validation experiments.

## 1. Introduction

Under cyclic loading and due to corrosive service environments, many high-speed rail components are likely to develop cracks. Cracks can affect the performance of the entire component, and security incidents can occur. As an effect of real-time crack diagnosis and monitoring, structural health monitoring (SHM) has been a hot area of high-speed rail research in recent years. The early detection of these cracks is a key element for ensuring the safety and functionality of high-speed rail structures [1,2]. Since Lamb waves can travel large distances with little attenuation, and are sensitive to initial cracks [3,4,5,6,7], the SHM method based on Lamb waves has become an effective and promising methodology in detecting cracks on plate-like structures [8,9].

The fundamental concept behind crack detection is that the features of Lamb waves, such as energy and waveform, will be altered by cracks in their path. Ihn and Chang [10,11] used a piezoelectric sensor and actuator network to generate and receive signals, respectively. Various cracks were diagnosed by comparing the energy of the damaged signal with that of the undamaged signal. Lu et al. [3] investigated the variation of reflection and transmission of Lamb wave energy under different crack lengths and angles through finite element simulation. Experiments were carried out to verify the correctness of the finite element simulation, and Hilbert transform was used to obtain Lamb wave energy. Liu et al. [12] used continuous wavelet transform (CWT) to extract features called energy ratio changes. On this basis, an optimized sensing network was studied, and an intelligent diagnosis system of structural cracks was built. Dao [13] proposed a structural crack detection method using cointegration and fractal signal processing to remove undesired disturbance variables such as temperature variations. Crack-sensitive features were isolated, and damage was effectively detected. Wang [14] developed a synthetic time-reversal method to overcome the limitation of the conventional phased array method under a pulse-echo model, and provided an efficient imaging method for crack detection and evaluation.

The method of crack detectability based on Lamb waves is sensitive to the waveform, frequency, and time duration of the excitation signal. In many studies on crack detection, a 3.5 or 5 cycle Hanning-windowed tone burst was commonly employed as an excitation waveform [15,16]. Chen [17] proposed a Lamb wave-particle filter (LW-PF)-based method for on-line fatigue crack detection, which uses a particle filter to deal with the crack evolution and monitoring uncertainties. Wang [18] proposed a nonlinear ultrasonic technology based on crack–wave interaction to investigate the growth of a fatigue crack. Yang [9] used the Bayesian updating method to update model parameters, in order to reduce the number of training samples, and reduce the prediction error. Although these waveforms maintained a satisfactory balance between good dispersion characteristics, mode purity, and time resolution, some challenges remained for crack detection. First, the time duration of the received signal is much longer than that of excitation during the propagation, which can increase the difficulty of signal processing, and reduce the resolution [19]. Moreover, due to the dispersion characteristics, the frequency of excitation of Lamb waves is limited to a low range, which reduces the sensitivity to the initial crack. By designing an excitation waveform, there is a feasible way to reduce the difficulty of signal processing, and improve the resolution. Lin et al. [20] proposed an excitation waveform design strategy in which any signal with δ-like autocorrelation could be employed to actuate a Lamb wave. High-resolution excitation waveforms, such as the linear chirp (L-Chirp) signal, nonlinear chirp (NL-Chirp) signal [21], Barker code (BC) [22], and Golay complementary code (GCC), were employed to accurately create the Lamb wave. With pulse compression technique, the received signal had a shorter time duration, and a higher resolution. Taking advantage of the high output resolution, these excitation waveforms have great potential for improving the accuracy of crack detection.

Despite the performance of these excitation waveforms being verified on the non-damaged plate structure, there are still some challenges remaining for damage detection. Reliable acquisition and interpretation of high-resolution Lamb wave signals is not a trivial task. The difficulty in the signal excitation and analysis lies in several aspects: (1) different structure geometries and damage types require different excitation waveforms, which are determined by parameters, such as the number of periods and frequency range. Therefore, the parameter optimization research of high-resolution waveform is required, as the key issues for crack damage detection.; (2) Compared with Hanning-windowed tone burst signals, the more complex waveforms and wider frequency range create significant difficulties in analyzing the high-resolution Lamb wave propagation. The FE simulation and experimental study are subjected to realizing and verifying the crack detection process; (3) the physical model which describes the relationship between damage sensitive features and the crack size needs highly specialized knowledge. An integrated method using high-resolution Lamb waves to detect and quantificationally predict crack growth should be developed. In addition, an excited Lamb wave signal is always limited to a low frequency, due to dispersion characteristics and multiple mode characteristics. However, the initial crack is more sensitive to an excitation signal with higher frequency [23]. To address this issue, dispersion compensation techniques have been applied to ensure inspection integrity. The pulse compression technique is a traditional radar signal processing technology that can observably improve resolution [24,25]. Combined with pulse compression, a high-resolution Lamb wave can obtain a wider frequency range than a tone burst signal [4]. On the other hand, the interaction mechanism between high-resolution Lamb waves and cracks is complex, so it is difficult to establish the relationship between them directly. A feasible method is to extract significant features from Lamb wave signals, and to establish a relationship model between features and crack length. In order to eliminate interference and improve accuracy, multiple features are usually used to establish the diagnostic model.

In this paper, combined with high-resolution Lamb waves, a novel strategy of crack damage quantification is proposed. The L-Chirp signal and GCC signal are used as the Lamb wave excitation signals. Numerical analysis and finite element simulation are used to determine the cycle and frequency parameter of excitation signals. The crack length is correlated with the damage features of high-resolution Lamb waves through signal processing. Both numerical and experimental studies are employed to verify the efficiency and accuracy. The rest of the study is structured as follows: in Section 2, the theory of the Lamb wave pulse compression technique and the high-resolution signals are introduced; in Section 3, different excitation waveform parameters, include cycle and frequency, are investigated by numerical analysis and finite element simulation; in Section 4, a series of simulations are performed using the L-Chirp signal and GCC signal as excitation waveforms. The output waveforms of the simulations are processed, and three features which change with crack length are extracted. With these features, a crack quantification model is established, and the probability of detection (POD) is used to verify the performance of the quantification model constructed with the simulation data. In addition, validation experiments are carried out in Section 5. Finally, conclusions are offered in Section 6.

## 2. Theory of High-Resolution Lamb Waveform Design

### 2.1. Theory of Lamb Wave Pulse Compression

A Lamb wave with excitation waveform *s*(*t*) spreads from actuator to receiver; the response signal can be written as

(1) $$r\left(t\right)=\frac{1}{2\pi }\overset{\infty }{\underset{-\infty }{\int }}S\left(\omega \right)H\left(\omega \right)e^{i\left(\omega t-kx\right)}d\omega$$

where *S*(*ω*) is the Fourier transform of *s*(*t*), *k* is the wave number which can reflect the dispersion characteristics, and *x* is the distance propagated.

By cross-correlating the response signal *r*(*t*) with the time-reversal excitation signal $\bar{s\left(t\right)}$, the pulse compression signal *c*(*t*) is given by:

(2) $$\begin{array}{cc} c\left(t\right) & =r\left(t\right)\;conv.\;\bar{s\left(t\right)}\\ & =\frac{1}{2\pi }\overset{\infty }{\underset{-\infty }{\int }}S\left(\omega \right)H\left(\omega \right)S{\left(\omega \right)}^{*}e^{i\omega t}e^{-ikx}d\omega \\ & =\frac{1}{2\pi }\overset{\infty }{\underset{-\infty }{\int }}{\left|S\left(\omega \right)\right|}^{2}H\left(\omega \right)e^{i\omega t}e^{-ikx}d\omega \end{array}$$

where *conv.* refers to the convolution process, the * refers to the complex conjugate, and *|S*(*ω*)*|*^2^ represents the Fourier transform of the autocorrelation function of *s*(*t*).

With dispersion item *e^-ikx^*, the change in the system’s structure cannot be observed directly in *c*(*t*), and dispersion must be removed. There are several methods to solve this issue in [26,27,28]. The one based on distance domain mapping is used in this paper. With dispersion compensation, the processed signal can be represented as:

(3) $$g\left(t\right)=\frac{1}{2\pi }\overset{\infty }{\underset{-\infty }{\int }}{\left|S\left(\omega \right)\right|}^{2}H\left(\omega \right)e^{i\omega t}d\omega$$

If the autocorrelation function of the excitation signal is similar to the δ-like function, Equation (3) becomes:

(4) g(t)≈h(t)

where *h*(*t*) is the inverse Fourier transform of H(ω), and the processed signal *g*(*t*) can change significantly with crack growth.

### 2.2. Excitation Waveform Design

According to Lin [4], the autocorrelation functions of four typical signals, L-Chirp, NL-Chirp, BC, and GCC, are δ-like functions. These excitation signals can receive better resolution after pulse compression. L-Chirp and GCC perform better than BC and NL-Chirp in terms of the autocorrelation curve. Due to their smallest main lobe width and smaller side lobe width, L-Chirp and GCC are chosen as excitation waveforms in this paper.

#### 2.2.1. L-Chirp Signal

A chirp signal is a type of sinusoidal signal, where the phase is a function of time. For the L-Chirp signal, the frequency is linearly swept from the lower frequency bound to the upper frequency in excitation time *t* ∈ [0, *T*], and the L-Chirp signal can be written as:

(5) $$s(t)=\sin\left(2\pi f_{0}t+\frac{\pi Bt^{2}}{T}\right)$$

where *f*_0_ is the lower frequency bound, *B* is the bandwidth, and *T* is the time duration of the chirp.

To control the signal cycle as a traditional tone burst signal, the phase function should satisfy the following equation:

(6) $$2\pi f_{0}T+\frac{\pi BT^{2}}{T}=2\pi N$$

where *N* is number of cycles. Then, substituting Equation (6) into Equation (5), the L-Chirp signal with controlled cycles can be written as:

(7) $$s(t)=\sin\left(2\pi f_{0}t+\frac{\left(2f_{0}+B\right)\pi Bt^{2}}{2N}\right),t\in [0,T]$$

**Figure 1:**
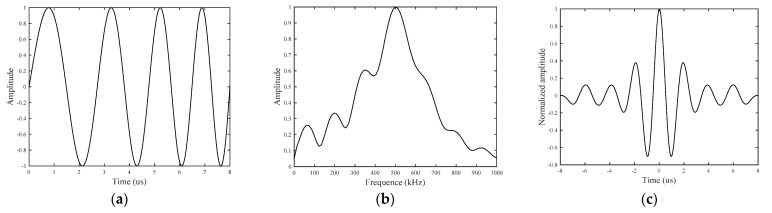
High-frequency L-Chirp signal: (**a**) waveform; (**b**) frequency spectrum; and (**c**) autocorrelation curve.

By defining *f*_0_ = 300 kHz, *B* = 400 kHz, and *N* = 4, the waveform, frequency spectrum, and autocorrelation curve of the waveform are shown in Figure 1.

#### 2.2.2. Golay Complementary Code

The GCC consists of two binary sequences:

(8) $$\begin{array}{l} A[N]=[a_{0},a_{1},a_{2},\cdots ,a_{N-1}],a_{i}\in \{-1,+1\}\\ B[N]=[b_{0},b_{1},b_{2},\cdots ,b_{N-1}],b_{i}\in \{-1,+1\}. \end{array}$$

In theory, the autocorrelation function of one binary sequence can be represented as:

(9) $$\Psi =\{\begin{array}{cc} N, & n=0\\ 0\;\mathrm{or}\;\pm 1, & n\neq 0 \end{array}$$

The autocorrelation functions of *A*[*N*] and *B*[*N*] have opposite side lobes. Therefore, the sum of the two autocorrelation functions is given by:

(10) $$\Psi_{AA}+\Psi_{BB}=\{\begin{array}{cc} 2N, & n=0\\ 0, & n\neq 0 \end{array}$$

In practice, the GCC cannot be directly used as an excitation signal. Usually, binary sequences are used as phase shift keys to modulate signals. In this paper, each code bit is modulated with a sinusoidal signal. In particular, +1 corresponds to a sinusoidal signal without an additional phase shift, and −1 corresponds to a sinusoidal signal with an additional 180° phase shift. In this paper, every code bit modulates one cycle sinusoidal signal in the excitation signal.

**Figure 2:**
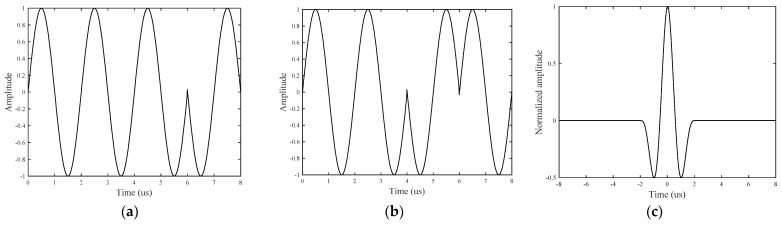
GCC signal: (**a**) sequence A; (**b**) sequence B; and (**c**) autocorrelation curve.

The GCC can lengthen without limit by recursively operating on a shorter GCC with the “negate and concatenate” method [4]. In practice, the GCC can be lengthened without limit by recursive repetition. Sequences A and B of the 4-bit GCC with a 500 kHz frequency sinusoidal signal are shown in Figure 2a,b, and the sum of the two autocorrelation functions is shown in Figure 2c.

## 3. Excitation Waveform Design for Crack Detection

Existing literatures focus on excitation waveforms design to achieve a better δ-like autocorrelation; only the performance of these excitation waveforms is verified on the non-damaged plate structure [4]. Nevertheless, the accuracy and efficiency of crack determination is determined by parameters such as the number of periods, and frequency range. When the time duration of a wave is longer, the time resolution is lower. Therefore, there is always a trade-off between a good dispersion characteristic and time resolution. Based on the dispersion characteristics of guided waves propagated in a thin plate, the sensitivity to structural flaws depends on the frequency range [23]. Therefore, the parameter optimization research of high-resolution waveforms for crack detection is studied in this paper.

### 3.1. Number of Periods

**Figure 3:**
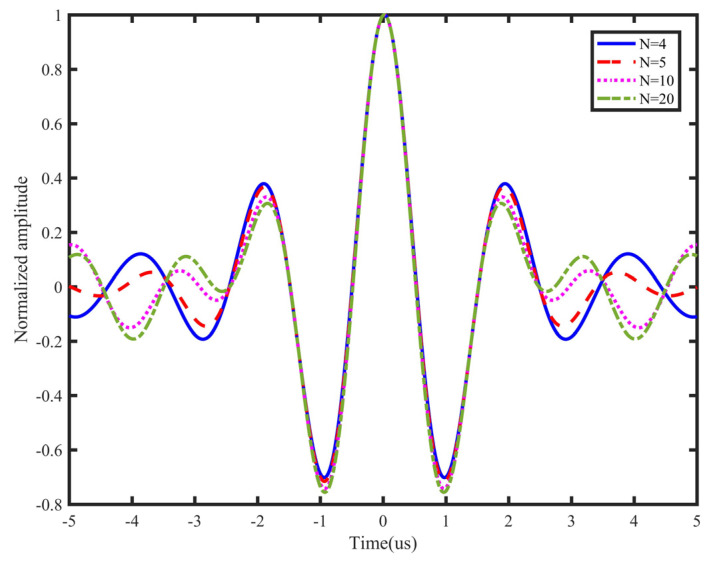
Autocorrelation curves of L-Chirp signals with different cycles.

The main lobe width and side lobe level of autocorrelation curves are employed as the criteria for choosing the number of periods [4,20]. With different numbers of cycles, the autocorrelation curves of 300~700 kHz L-Chip signals are shown in Figure 3. It can be easily observed that the number of cycles does not influence the main lobe width; moreover, it only slightly influences the side lobe level. Therefore, using L-Chirp signals with different numbers of cycles creates the same effects. To avoid overlapping the directly received signal with the boundary reflection signal, and to increase the calculation speed, four-cycle L-Chirp signals are used as excitation signals in this paper.

**Figure 4:**
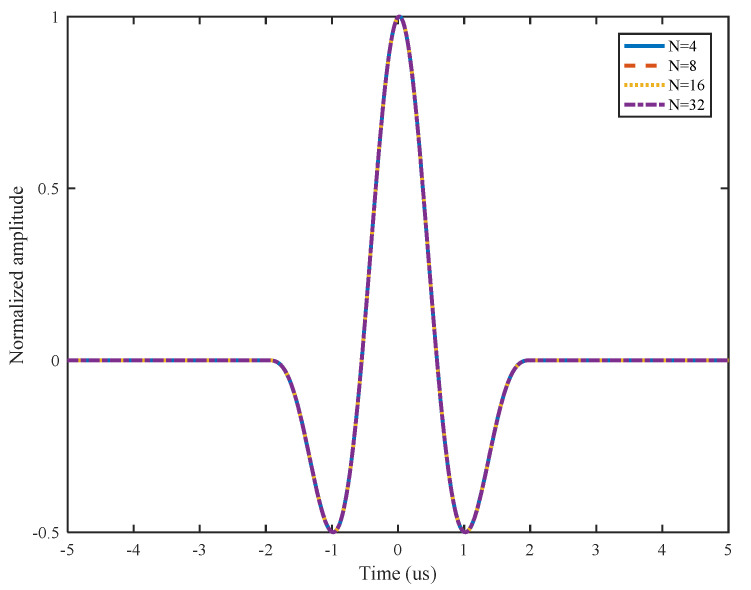
Autocorrelation curves of GCC signals with different numbers of cycles.

As discussed in the L-Chirp signal, the autocorrelation curves of GCC signals with different numbers of cycles are shown in Figure 4. All of the autocorrelation curves overlap, and there is the same effect using GCC signals with different numbers of cycles. Hence, the 4-bit GCC signal is chosen as the excitation signal, to avoid overlapping signals and increase the calculation speed.

### 3.2. Frequency Range

Depending on the dispersion characteristic of the Lamb wave, the frequency-thickness values are kept below the cut-off frequency, where there is only S_0_ mode and A_0_ mode. The frequency–thickness value is always limited to 1 Mhz∙mm, to avoid mode superposition. Combined with pulse compression, a high-resolution Lamb wave can obtain a wider frequency range than a tone burst signal, which improves the sensitivity to the initial crack. The excitation frequency range of L-Chirp and GCC are investigated though FE simulation in this paper.

**Figure 5:**
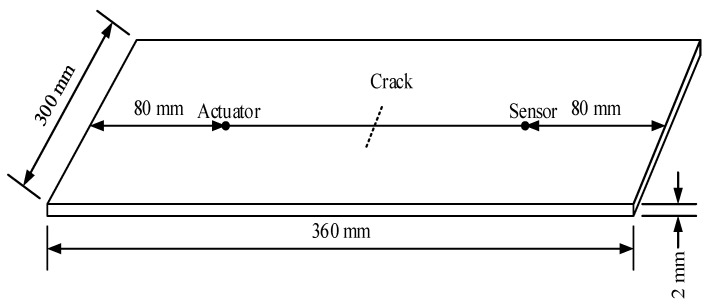
Dimension of the plate structure and crack location.

The specimen for the simulation is made of 2024-T3 aluminum, the detailed geometry of which is shown in Figure 5, and the mechanical properties are listed in Table 1. A through-thickness center crack with a 0.3 mm width is prefabricated. An actuator and a receiver are placed on either side of the crack as a pitch-catch configuration. The distance between them is 200 mm. In order to compare the performance of signals with different frequencies, 0~400 kHz, 100~500 kHz, 200~600 kHz, and 300~700 kHz L-Chirp signals, and 200 kHz, 300 kHz, 400 kHz, and 500 kHz GCC signals are used as excitation waveforms. In this paper, the ability of detecting initial cracks is paid more attention. Therefore, the length of the simulation crack is set to 0 mm, 0.5 mm, 1 mm, 1.5 mm, and 2 mm. To maintain the accuracy of simulations, the element length should be in tune with the time step of the simulation. As such, the propagating waves can spatially be resolved. Hence, it is necessary to set up more than 10 nodes per wavelength. The smallest wavelength of the chosen high-resolution Lamb waves is 4.46 mm [16,29]. The element length is set to 0.2 mm. The time step is set to 0.01 μs.

It is known that the S_0_ mode of a Lamb wave is more sensitive to cracks than A_0_ mode [30]. Taking the advantage of faster group velocity, the first received S_0_ mode can be easily distinguished. As mentioned earlier, the dispersive nature of Lamb waves makes it difficult to extract features of fatigue crack damage, and reduces the resolution. The dispersion compensation is applied to eliminate the effect of dispersion in the numerical simulation. Meanwhile, the pulse compression technique is used to improve the resolution, and make the processed waveform change more obvious in the damaged structure. In this paper, the S_0_ direct wave packets after pulse compression in these responses are extracted for analysis.

**Figure 6:**
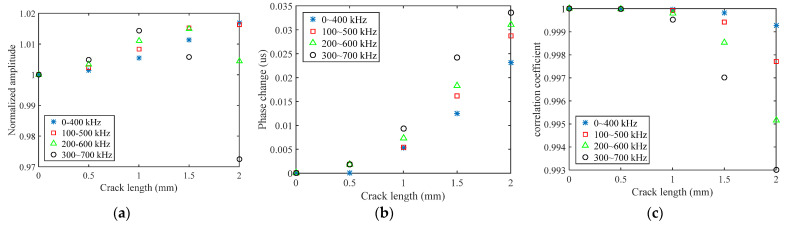
Crack length versus damage features of L-Chirp signals: (**a**) normalized amplitude; (**b**) phase change; (**c**) correlation coefficient.

**Figure 7:**
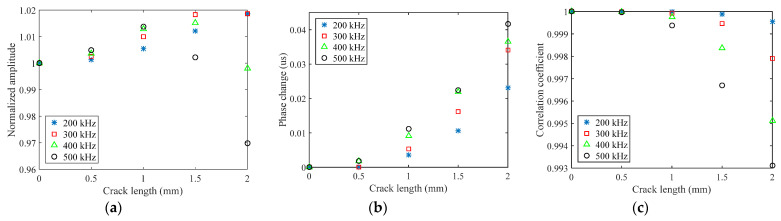
Crack length versus damage features of GCC signals: (**a**) normalized amplitude; (**b**) phase change; (**c**) correlation coefficient.

Direct use of the processed signal for the choice of frequency range is difficult, and data reduction is generally required to extract the damage feature. Three damage features are extracted in this paper, namely normalized amplitude, phase change, and the correlation coefficient. The amplitude of received signal data decreases with the increase of crack length, which may be due to reflection and scattering. A normalized amplitude is defined as the ratio of a damaged signal amplitude to a healthy signal amplitude, which is used to remove the effects of uncertainties from differences in soldering and bonding. Considering an open crack under tension loading, only the scattered waves in a detour route from the crack tip are collected. The detour route is extracted as a phase change. In this way, the arrival time of the S_0_ direct wave packets will be different between the plates with and without crack. If there is damage that is located on or is close to the sensing path, the received signal would change dramatically, and the correlation coefficient between the two signals would be relatively small. It should be noted that the correlation coefficient is sensitive to the signal phase and waveform. The crack length versus the normalized amplitude, phase change, and correlation coefficient for different excitation waveforms are shown in Figure 6 and Figure 7. The phase change increases as the crack length increases; on the contrary, the normalized amplitude and correlation coefficient decrease as the crack length increases. It is also observed that each excitation waveform has a unique trend. For the correlation coefficient and phase change, either L-Chirp or GCC with a higher frequency range have a higher absolute value of slope than others, which means that these damaged features extracted from a higher frequency signal are more sensitive to the initial crack. When there is a hairline crack, the signals collected by the sensor include the transmitted wave signals travelling across the crack, and the scattered wave signals originating from the crack tip, resulting in an increase in the amplitude [16]. Therefore, the normalized amplitude increases slightly, and subsequently decreases versus the crack length, as shown in Figure 6a and Figure 7a. Nevertheless, the normalized amplitude is generally assumed to be monotonically decreasing in crack detection. In terms of a crack quantification model, the shorter normalized amplitude rising region of the excitation waveforms with higher frequency will lead a better crack detection result. Hence, 300~700 kHz L-Chirp signals and 500 kHz GCC signals are used as excitation waveforms in numerical simulations and experimental study. 

## 4. Detection Model Establishment

### 4.1. High-Resolution Lamb Wave Simulation

**Figure 8:**
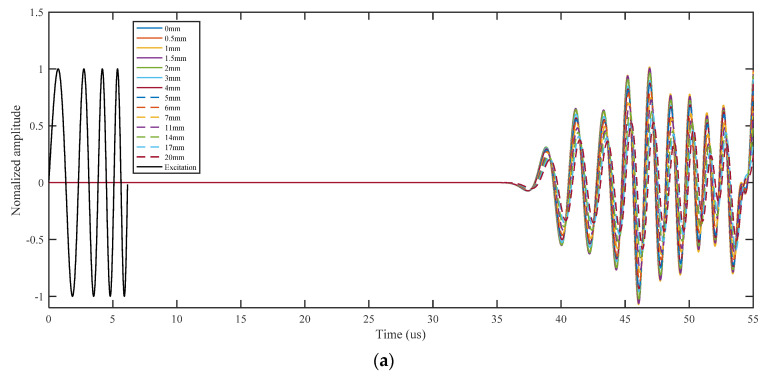

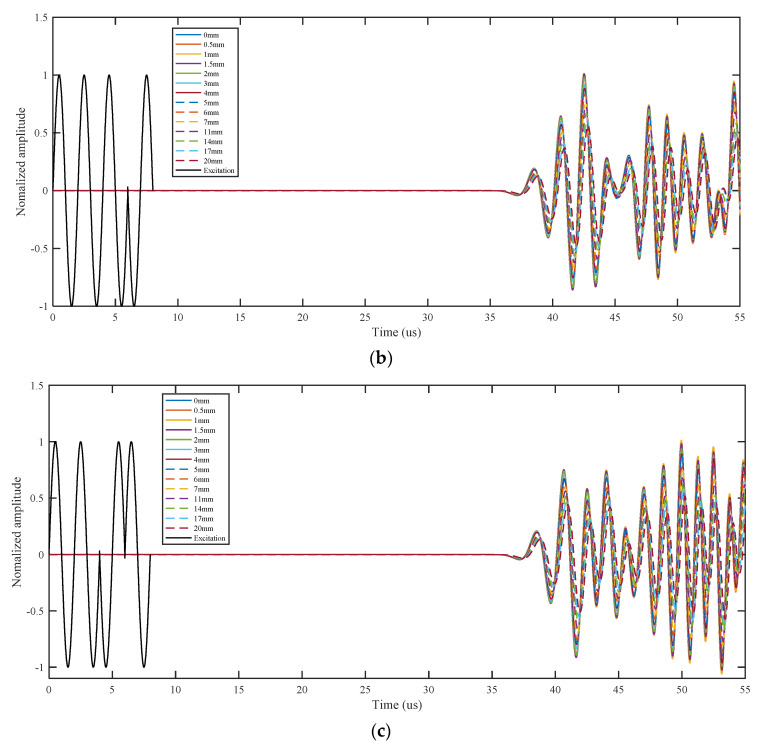
FE simulation of Lamb wave response with different crack lengths: (**a**) L-Chip signal; (**b**) A wave of GCC signal; (**c**) B-wave of GCC signal.

Compared with Hanning-windowed tone burst signals, the more complex waveforms and wider frequency range cause significant difficulties in analyzing the high-resolution Lamb wave propagation. The finite element (FE) simulation is widely used to analyze the damage detection process based on Lamb waves. The high-resolution Lamb wave responses of crack damages are simulated to realize and verify the proposed approach. The simulation model is same the model in Section 2 as shown in Figure 5. In this paper, a 0~300 kHz L-Chirp signal, a 150 kHz GCC signal, a 300~700 kHz L-Chirp signal, and a 500 kHz GCC signal are used as excitations. The length of the crack is set from 0 mm to 20 mm. FE simulation results of different excitation waveforms are obtained, and shown in Figure 8. The data from the plate without any crack and the data with different crack sizes are presented for comparison purposes. It is observed that the time duration of the output signals much larger than that of the excitation, which increases the difficulties of signal interpretation. This phenomenon is more obvious in L-Chirp, due to the larger frequency range. The pulse compression method is designed and used for resolution enhancement in the following:

### 4.2. Signal Processing

**Figure 9:**
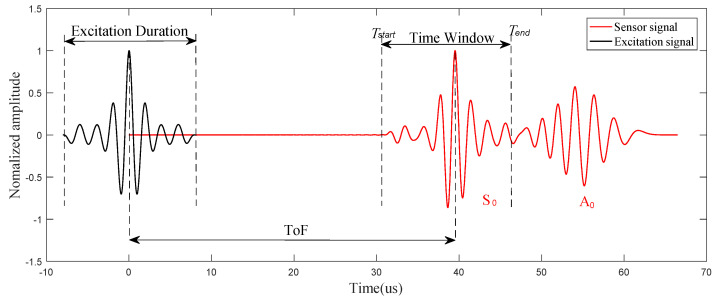
Schematic illustration for the time window calculation (300~700 kHz L-Chip).

**Figure 10:**
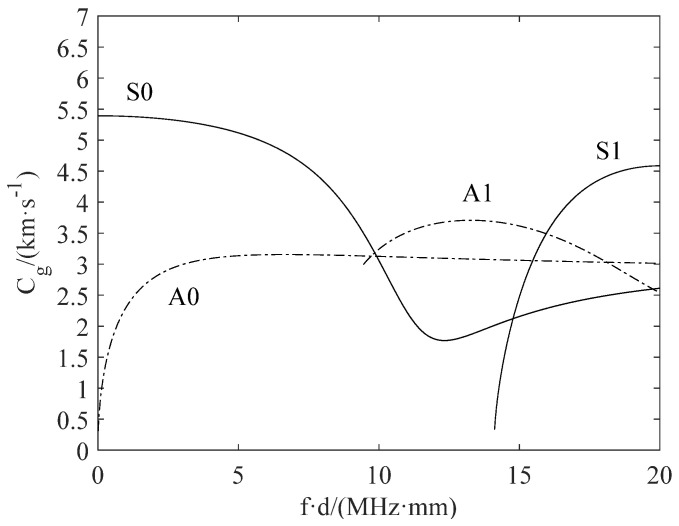
The dispersion curve of 2024 aluminum.

**Figure 11:**
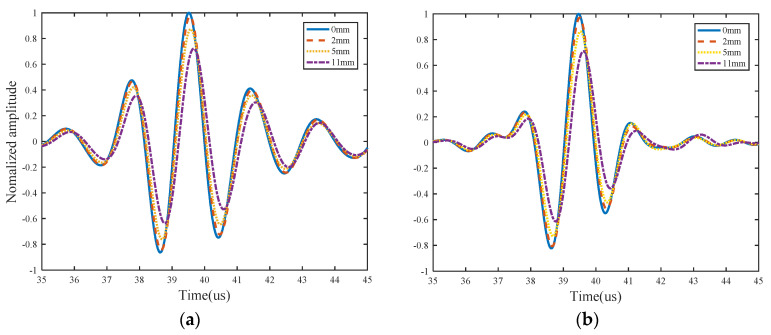
Some waveforms processed with dispersion compensation pulse compression of (**a**) 300~700 kHz L-Chirp signal and (**b**) 500 kHz GCC signal.

As mentioned earlier, the dispersive nature of Lamb waves causes difficulties in extracting features of fatigue crack damage, and reduces the resolution. The dispersion compensation is applied to eliminate the effect of dispersion in the numerical simulation. Meanwhile, the pulse compression technique is used to improve the resolution, and cause the processed waveform change to be more obvious in the damaged structure. Taking advantage of more sensitivity to crack damage, the S_0_ direct wave packets after pulse compression in these responses are extracted for analysis. The demonstration of time-of-flight (ToF) and the time window are shown in Figure 9. After the pulse compression process, the effects of dispersion are removed, and resolution of L-Chirp and GCC excitation waveforms is observably improved. The first S_0_ direct wave packets can be clearly distinguished in the figure. The group velocity of S_0_ mode is a key parameter to calculate the time window of first wave package in time-domain, which can be determined experimentally or analytically. In the numerical simulation, the group velocity of S_0_ mode is obtained in accordance with the dispersion curve. In the experimental study, the group velocity can be verified in an undamaged specimen by measuring the ToF between two sensors with a known distance. The group velocity of 300~700 kHz L-Chip and 500 kHz 309 GCC excitation signal are analytically obtained as 5063 m/s and 5115 m/s, respectively, and the group velocity is consistent with the theoretical velocity shown in Figure 10. In this paper, the time window of the S_0_ first wave package is defined as the time duration between the start time point and end time point, which is consistent with the length of the excitation duration. The response signal data clipped to the calculated time window after dispersion compensation and pulse compression are presented in Figure 11.

**Figure 12:**
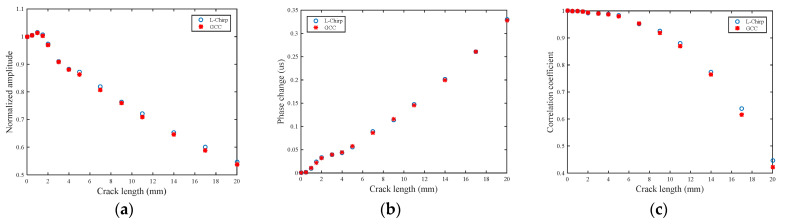
Damage features of the processed signal in the simulation: (**a**) normalized amplitude; (**b**) phase change; (**c**) correlation coefficient.

As mentioned in Section 3, three damage features are extracted to detect crack damage, namely normalized amplitude, phase change, and the correlation coefficient. The relationships between the crack length with these three damage features are shown in Figure 12. The trends can accord with the physics mechanisms analysis, which manifest that the proposed methods are reasonable. 

### 4.3. Crack Quantification Model

In accordance with the above analysis of damage sensitive features, a regression model is employed to describe the crack length and these characteristics:

(11) $$L=a_{0}+a_{1}x+a_{2}y+a_{3}z+a_{4}x^{2}+a_{5}y^{2}+a_{6}z^{2}$$

where *x* is the normalized amplitude, *y* is the phase change, *z* is the correlation coefficient, and *a*_0_~*a*_6_ are model parameters that can be determined using the Bayesian estimator method [16]. The regression model is not fixed. The other formulations can also be applied after investigation.

**Figure 13:**
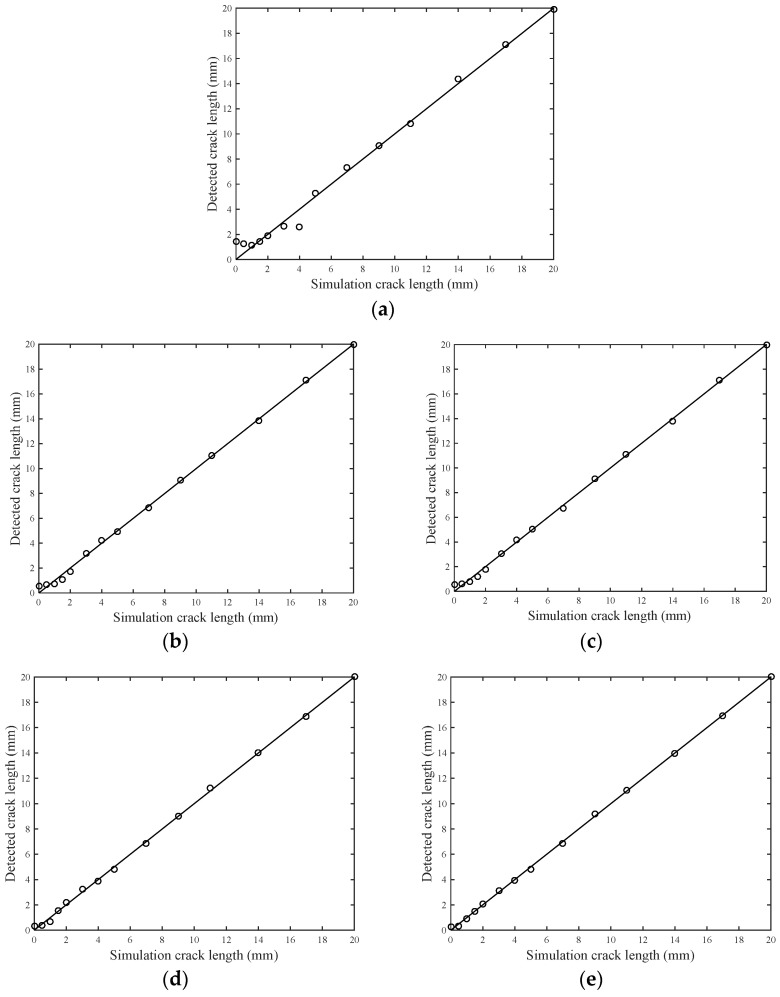
Fitting curves: (**a**) 3.5 cycle Hanning-windowed tone burst signal; (**b**) 0~300 kHz L-Chirp signal; (**c**) 150 kHz GCC signal; (**d**) 300~700 kHz L-Chirp signal; and (**e**) 500 kHz GCC signal.

To investigate the performance of traditional excitation waveforms and high-resolution excitation waveforms using the proposed multi-feature integration approach, a 3.5 cycle Hanning-windowed tone burst signal with a frequency of 160 Hz, a 0~300 kHz L-Chirp signal, and a 150 kHz GCC signal are used as an excitation waveform in an FE simulation, respectively. The Lamb wave excitation signal with higher frequency has, in particular, proven more susceptible to the tiniest change in the plate along or near the sensing paths, which is superior to other methods, in the aspect of sensitivity to the initial crack. Nevertheless, the traditional Lamb wave excitation signal is always limited to a low frequency range, due to dispersion characteristics. To circumvent this, a 300~700 kHz L-Chirp signal and a 500 kHz GCC signal are employed as high-frequency excitation waveforms in an FE simulation. In this study, the FE simulation results with different excitation waveforms are used to establish the corresponding multi-feature integration predictive model in terms of Equation (11). These three damage sensitive features are obtained from the receiving signal. Further, the Bayesian estimator method is employed to estimate the parameter, and the model parameters are shown in Table 2. The results of the five models are shown in Figure 13. Good agreements are observed. 

### 4.4. Model Performance Verification

The performances of different excitation waveforms are discussed by using POD and average error; the reliability of the detection model and the accuracy of the detection model are evaluated, respectively [31]. The POD model will be derived first. It can be assumed that logarithm relations exist between the detected crack size $\hat{L}$, and the actual crack length *L*, which is illustrated as:

(12) $$\ln\hat{L}=\alpha +\beta \ln L+\varepsilon$$

where *α* and *β* are parameters that can be determined using random sampling, and *ε* is a error term. Assuming that *ε* is a zero mean normal variable, the POD can be expressed as:

(13) $$POD\left(L\right)=P\left(\alpha +\beta \ln L+\varepsilon >\ln L_{th}\right)=\Phi \left(\frac{\ln L-\left(\ln L_{th}-\alpha \right)/\beta }{\sigma_{\varepsilon }/\beta }\right)$$

where *σ_ε_* is the standard deviation of the error term, *L_th_* is the minimum detectable size depend on the monitoring equipment and external environment, and Φ(·) is the standard normal cumulative distribution function.

Using the simulation result of different excitation waveforms, parameter estimation of the five models is carried out. The model performance in terms of average error, standard deviation, and R-square are presented in Table 3. It is obvious that the high-resolution yields a smaller average error and standard deviation, and a larger R-square. That is because the high-resolution excitation waveforms, combined with pulse compression, can improve the time resolution, and improve the accuracy of crack detection. With an increase in excitation frequency of L-Chirp and GCC waveforms, the more accurate crack size predictions are acquired. 

**Figure 14:**
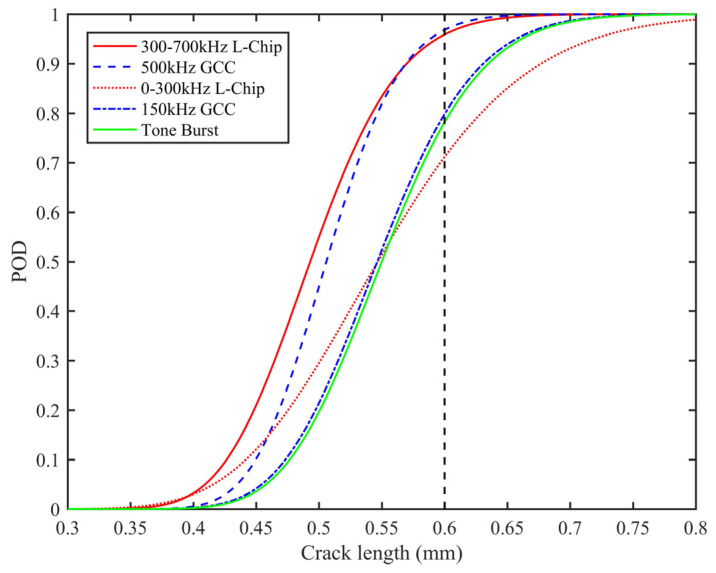
POD curves of different excitations.

To further examine the detection reliability of the high-resolution excitation waveforms, the data of the detected crack length and the actual crack length are used to achieve POD results. The average error is preferred for quantitative crack length calculation, while POD is more appropriate for small crack detection. The minimum detectable size is set to 0.6 mm in the POD model. Figure 14 shows the POD curves associated with the five excitation waveforms. When the crack size is less than 0.6 mm, 300~700 kHz L-chirp and 500 kHz GCC perform better than other waveforms. Accordingly, these excitation waveforms have a greater capacity to identify the initial crack, benefiting from the optimal design of the excitation waveform, the dispersion compensation and pulse compression techniques, and a higher selectable frequency range. When the crack length exceeds 0.8 mm, PODs of the five excitation waveforms are approximately equal to 100%. In contrast, although 0~300 kHz L-Chirp and 150 kHz GCC have a smaller average error than the Hanning-windowed tone burst, their POD performance does not outperform the tone burst waveforms. To summarize the above, 300~700 kHz L-chirp and 500 kHz GCC yield the best detection performance among all excitation waveforms, and are used as excitation waveforms in a later experiment.

## 5. Experimental Validation

Lamb wave testing using surface-bonded piezoelectric wafers is conducted to further verify the proposed method. In this testing, the aluminum plates are subjected to artificial cracks. The 300~700 kHz L-Chirp signal and 500 kHz GCC signal are set as excitation waveforms. The dispersion compensation and pulse compression techniques are applied to process the received signals to obtain high resolution. Three damage-sensitive features are extracted to establish a regression model to calculate the crack sizes.

### 5.1. Experimental Designs

**Figure 15:**
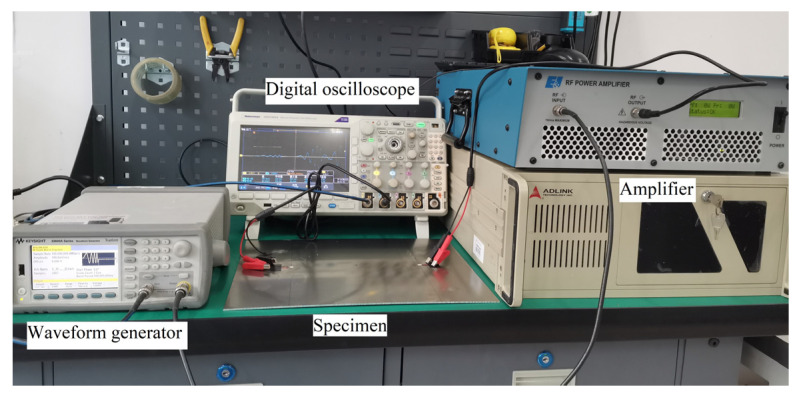
Experimental setup for coupon test.

The specimen is made of 2024 aluminum. The geometry, sensor layout, and mechanical properties of the specimen are the same as those of the simulation model in Section 3, which are shown in Figure 5 and Table 1. A through-thickness center crack whose width is 0.5 mm is prefabricated through electric discharge machining (EDM). Firstly, Lamb wave signals received from five intact specimens T0~T4 are obtained as the perfect state without damage. Then, the center crack is introduced by EDM from 0.5 mm to 15 mm, and the actual crack length is read by microscopy. The high-resolution excitation waveforms are generated by a KEYSIGHT 33600A series waveform generator. The excitation waveform was amplified 30 dB before it was sent to the specimens. A Tektronix MOD3024 multichannel digital oscilloscope is used for data collection. The experimental setup is shown in Figure 15.

**Figure 16:**
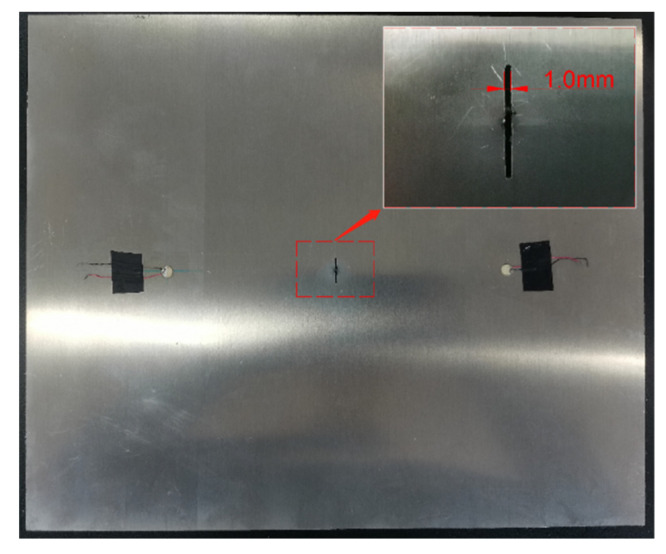
The diagram of crack width at 15 mm crack length.

For structural health monitoring in an aluminum alloy plate, the crack damage is generally considered as a notch or an open crack where there is no contact between two cracked surfaces [9,15,32,33]. In this study, Lamb wave testing is performed on plates with artificial cracks or simulation cracks of different sizes to characterize a through-thickness crack under tensile load. Thus, the depth of the crack is a constant equal to the thickness of the plate, measuring 2 mm. Moreover, it is of great significance to consider the influence of crack depth in composite laminates structures [34,35]. In experimental validation, a through-thickness crack is introduced through electric discharge machining (EDM), with a 0.5 mm initial width in the middle of the plate. Because each EDM process should expand the width of crack, the width of crack increased to 1 mm when the length of crack measured 15 mm, as shown in Figure 16. In this way, the influence of crack width is introduced in the crack detection model.

### 5.2. Crack Detection Results

**Figure 17:**
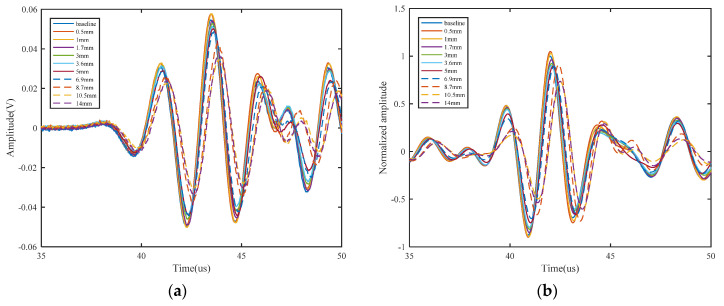
Received waveform of 300~700 kHz L-Chip signal: (**a**) raw signal; and (**b**) the signal processed by dispersion compensation and pulse compression techniques.

**Figure 18:**
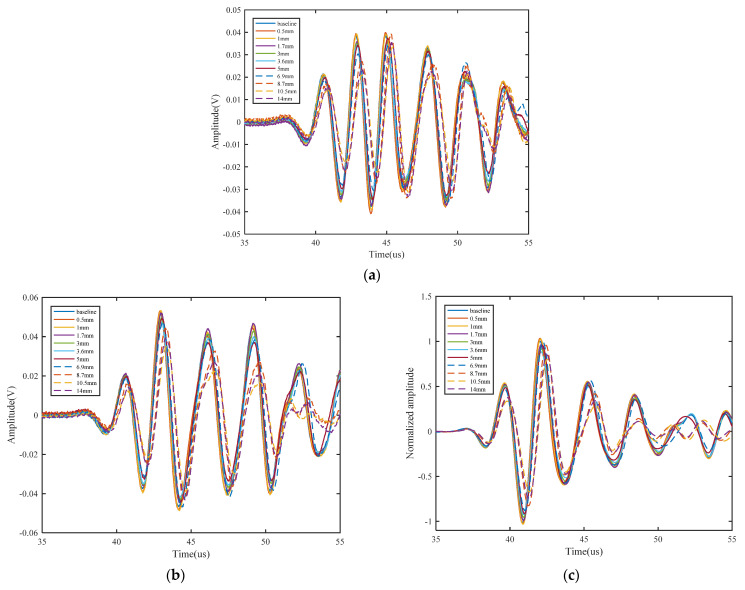
Received waveform of 500 kHz GCC signal: (**a**) raw signal of sequence A; (**b**) raw signal of sequence B; (**c**) the signal processed by dispersion compensation and pulse compression techniques.

**Figure 19:**
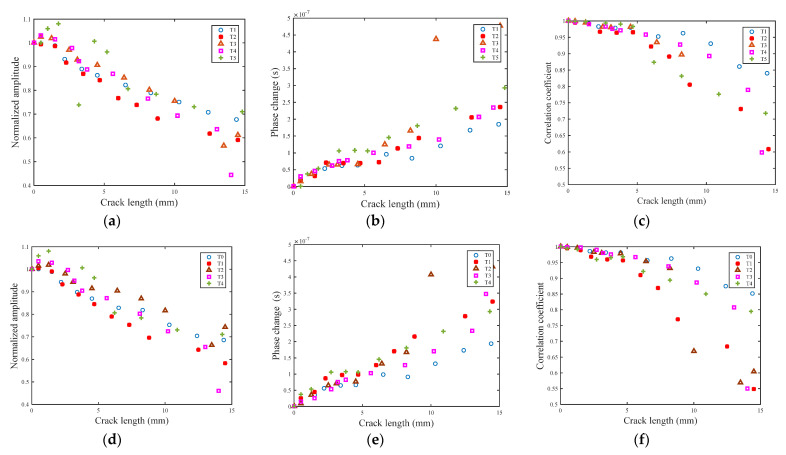
Damage features of specimens: (**a**) normalized amplitude of 300~700 kHz L-Chip; (**b**) phase change of 300~700 kHz L-Chip; (**c**) correlation coefficient of 300~700 kHz L-Chip; (**d**) normalized amplitude of 500 kHz GCC; (**e**) phase change of 500 kHz GCC; and (**f**) correlation coefficient of 500 kHz GCC.

The results of the healthy, non-damaged specimens are regarded as a baseline. By comparing this baseline with the received signal of damaged specimens, feature variation caused by cracks could be observed. With a 300~700 kHz L-Chip and 500 kHz GCC excitation signal, the raw received signals of specimen T1 are shown in Figure 17a and Figure 18a,b, respectively. To obtain high resolution, the dispersion compensation and pulse compression techniques are subjected to processing all received signals. The processed results are shown in Figure 17b and Figure 18c, respectively. After the pulse compression, the first wave packet of the S_0_ mode is chosen for feature extraction. The crack length versus three damage features are shown in Figure 19. There are monotonic variation trends between the features and crack length increment. In addition, the results across samples are consistent. The difference in the samples is caused by the small difference in plate geometry, and a coupling situation between the sensor and specimen.

**Figure 20:**
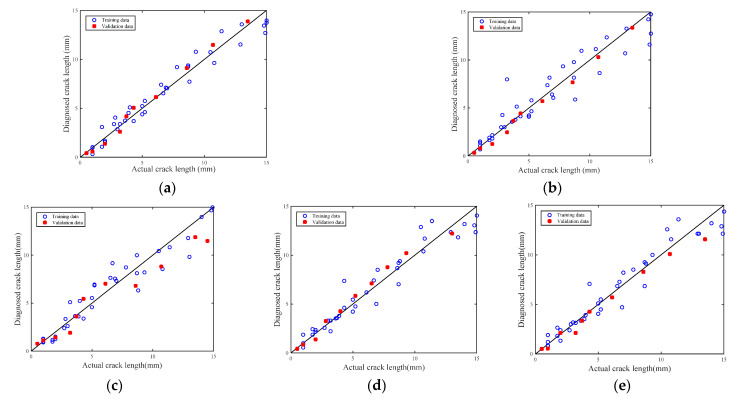
Diagnosed crack lengths with (**a**) 300–700 kHz L-Chip excitation signal; (**b**) 500 kHz GCC excitation signal; (**c**) 3.5 cycle 160 kHz Hanning-windowed tone burst signal; (**d**) 0–300 kHz L-Chirp excitation signal; and (**e**) 150 kHz GCC excitation signal.

A regression model, Equation (11), is used. In this study, the damage features and crack length of four specimens (T1, T2, T3 and T4) are used to calibrate the parameters. The data of specimen T0 are used for validations. The detected results of the 300~700 kHz L-Chip and 500 kHz GCC excitation waveforms are shown in Figure 20a,b. A good agreement can be observed. The average prediction error for the 300~700 kHz L-Chip and 500 kHz GCC are 0.4215 mm and 0.4005 mm, respectively. The R-square of these two models is 0.964686 and 0.917171, respectively. The maximum error of the 300~700 kHz L-Chip and 500 kHz GCC signal above a 5 mm crack length are 0.7628 mm and 0.9071 mm, and relative error are 3.90% and 5.86%, respectively. Compared with the traditional 3.5 cycle 160 kHz Hanning-windowed tone burst signal, the maximum error for a signal above a 5 mm crack length is 2.9994 mm, and relative error is 20.03%. Therefore, the error of predication of crack length using high-resolution Lamb waves is proved to be acceptable. In order to further verify the crack detection performance of the 300~700 kHz L-Chirp signal and 500 kHz GCC signal, the 3.5 cycle 160 kHz Hanning-windowed tone burst signal, 0~300 kHz L-Chirp signal, and 150 kHz GCC signal are also set as excitation signals for experiments. The experimental process and model establishment method are the same as that of the 300~700 kHz L-Chirp signal and 500 kHz GCC signal. The detection results of these three signals are shown in Figure 20c,d. The average prediction error for the 3.5 cycle Hanning-windowed tone burst signal, 0~300 kHz L-Chirp signal, and 150 kHz GCC signal are 0.9689 mm, 0.7316 mm, and 0.7250 mm, respectively. In summary, the crack detection performance of the 300~700 kHz L-Chirp signal and 500 kHz GCC signal is better than that of the 3.5 cycle Hanning-windowed tone burst signal, 0~300 kHz L-Chirp signal, and 150 kHz GCC signal.

## 6. Conclusions

This paper improved an integrated method using high-resolution Lamb waves to detect and quantificationally predict crack growth in a plate. The propagation mechanisms and the interaction with defects of several typical signals (including L-Chirp and GCC) are studied through numerical simulation. A strategy for the number of periods and frequency range determination is established. The dispersion compensation and the pulse compression are subject to eliminating dispersion characteristics, and attaining a high output resolution. Three damage features, namely normalized amplitude, phase change, and the correlation coefficient, are extracted from the first S_0_ wave packet, which has an explicit physics explanation. Furthermore, a multi-feature integration method is proposed to compute crack size. The crack quantification performance of the designed waveforms and traditional tone burst is verified. 

Based on a theoretical study, numerical simulations, and validation experiments, some conclusions can be obtained as follows:

1. The parameter optimization of high-resolution waveforms for crack detection is studied, and a strategy for the number of periods and frequency range determination is established. The high-resolution Lamb wave can obtain a wider frequency range than a tone burst signal, which is beneficial for initial crack detection.
2. The pulse compression technique is used to improve the resolution and make the processed waveform change more obvious in the damaged structure. Three damage features, namely normalized amplitude, phase change, and the correlation coefficient, are extracted from the S_0_ direct wave packets after pulse compression. The regression model using these three features yield satisfactory prediction results.
3. The numerical studies and experimental validation are both conducted on the aluminum alloy plate for verification. The results illustrate that L-Chirp and GCC with higher frequency have a greater capacity for identifying both the initial crack and longer crack size than the traditional Hanning-widowed tone burst.

It should be noted that the crack damage location is fixed in the middle of the plate, and the crack orientation is perpendicular to the Lamb wave propagation paths in this paper. For engineering practice, the crack orientation is a critical parameter which has a significant influence on Lamb wave propagation. In such a case, the proposed crack detection method based on high-resolution Lamb waves cannot directly applied. Future studies should be focused on quantitative relationships between the reflection/transmission coefficient and crack orientation. In addition, using some intelligent algorithms to extract more damage features might be identified to enhance the accuracy and robustness of the crack detection model. The applicability of the proposed method to other structural and material systems, such as composite skins, also needs further investigation. More coding signals can be investigated as excitation Lamb waveforms for damage identification in both composite and metallic materials in the furfure, such as Gold code [36], and M sequence code [37].

## Figures and Tables

**Table 1:** Mechanical properties.

Material	*E* (GPa)	*v*	*ρ* (kg/m^3^)
Al2024-T3	72	0.33	2780

**Table 2:** The model parameters of 300~700 kHz L-Chirp and 500 kHz GCC.

Parameters	Tone Burst Signal	0~300 kHz L-Chirp	150 kHz GCC	300~700 kHz L-Chirp	500 kHz GCC
a_0_	−460.12	−57.25	−534.66	−17.13	−17.53
a_1_	−355.22	−352.10	−369.86	−103.54	−139.76
a_2_	27.30	39.13	19.44	54.82	62.88
a_3_	1624.77	451.29	1947.6	72.65	85.82
a_4_	203.56	182.51	215.82	47.64	68.12
a_5_	−121.39	−28.03	−149.40	264.91	329.85
a_6_	−1233.09	−223.91	−1258.43	0.69	3.59

**Table 3:** Average error and standard deviation of different excitations.

Excitation Waveform	Tone Burst	0~300 kHz L-Chirp	150 kHz GCC	300~700 kHz L-Chirp	500 kHz GCC
average error (mm)	0.3282	0.1941	0.1855	0.0886	0.0676
Standard deviation (mm)	0.5246	0.2443	0.2332	0.1187	0.0880
R-square	0.992852	0.998586	0.998711	0.999225	0.999621

## Data Availability

Not applicable.
